# Supplementary material for: Analysis of Food Safety Management Systems in the Beef Meat Processing and Distribution Chain in Uganda
Source: Foods. 2021 Sep 22;10(10):2244. doi: 10.3390/foods10102244 (PMC8534317; doi:10.3390/foods10102244)
Supplement: Supplementary file 1 [file foods-10-02244-s001.zip › foods-1333395-supplementary.pdf]

Interview Questions – Butchershops and Supermarkets

<u>Part I: Backward linkages (relationship with the slaughterhouses)</u>

1. Describe the company's contractual relationship with the slaughterhouses
   1.1. How many slaughterhouses did you use to buy meat during the previous year? __________
   1.2. If you used more than one slaughterhouse during the previous year, what was the main reason for doing so? 1= To search for a better price 2= To search for good quality meat for my customers 3= Due to safety concerns 4= Other (specify) _____________________________
   1.3. If you had used only one slaughterhouse during the previous year, what was the main reason for doing so? 1= We have a long-term contract with the slaughterhouse 2= This slaughterhouse always offers a better price than others 2= This slaughterhouse supplies good quality meat at all times 3= This slaughterhouse supplies safe meat (is certified by third-party) 4= Other (specify) _____________________________
   1.4. Considering the **<u>past few years</u>**, which slaughterhouses did you use **<u>most</u>** as a source of meat for your butcher shop? 1= Nsooba slaughterhouse Ltd 2= City Abattoir 3= Ugandan Meat Packers Ltd 4=Other
   1.5. *Think of the slaughterhouse that <u>supplied most of the meat during the past few years</u>, and answer the following questions <u>only based on your relationship with</u> <u>this slaughterhouse</u>.*
      1.5.1. Duration of relationship: How long have you been buying meat from this slaughterhouse? _______ (years)
      1.5.2. What is the nature of communication between your company and the slaughterhouse? 1=There is always a two-way communication between the company and this slaughterhouse; 2= Two-way communication is occasional or none – existence.
      1.5.3. What type of information do you often exchange with the slaughterhouse *(more than one answer is possible)*? 1=Product information exchange (e.g., animal feed, type of breed, the animal origin/location); 2= Process information exchange (e.g., laboratory results of microbial levels and pathogens, hygiene, traceability, and safety, etc.); 3= Planning information (Demand forecasts, quantity, quality, etc.); 4= Other (specify)
      1.5.4. The formality of exchange: 1= The communication between my company and this slaughterhouse is mostly formal (i.e., in writing or by email); 2= Informal (phone calls or visits)

1.5.5. The nature of contract terms: 1= ***Closed one*** – i.e., the contract terms related to price, delivery, quality and risks (safety conditions) are pre-agreed and documented (in writing), and cannot be modified in the event of unexpected market conditions; 2= ***Flexible*** – i.e., the contract terms related to price, delivery, quality and risks (safety conditions) are pre-agreed and documented (in writing), but are subject to modifications in the event of unexpected market conditions ; 3= ***Relational*** – the contract terms related to price, delivery, quality and risks (safety conditions) are pre-agreed **but not in written form** and are subject to modification depending on the market conditions; 4=

1.5.6. What percent of the total meat delivered by this slaughterhouse is being rejected by the company on average per year? _______________

1.5.7. Have you ever had incidences of detecting excessive microbial pathogens from the meat supplied by this slaughterhouse in the previous one year? 1=Yes 2= No, 3= We don't know because we don't have the laboratory/facility to test microbial pathogen levels

2. In your opinion, what are the most important factors affecting the **safety/quality of meat** in Uganda? Please mention up to three.

2.1. First most important factor ____

2.2. Second most important facto ____

2.3. Third most important factor ____

Part 2: Assessing the food safety control system implemented by the butcher shops/supermarkets

1. Does your company have any Quality Assurance standards/guidelines (e.g. GMP, GHP, HACCP, ISO 9001, ISO 22000 that have been implemented? 1=Yes 2= No

2. **Chose which option(s) best describes what you do at your butcher shop**

2.1. Pest control (Rodents, cockroaches, and flies)

2.1.1. What are the control measures you carry out in order to overcome issues related to pests?

a. Presence of rodent traps like a rat trap, housefly traps, fly screens, etc. 1= Yes; 2=No
b. Keeping the floors, walls, roof, doors and window openings in a good state of repair with no gaps or spaces. = Yes; 2=No
c. keeping the meat in pest-proof containers. 1= Yes; 2=No
d. Use of electronic fly devices. 1= Yes; 2=No
e. Others. Specify…………

2.1.2. How often do you check for signs of pests such as rodent droppings, smear marks, insect egg cases, and either live or dead insects done? 1=daily; 2=weekly; 3=monthly; 4=annually; 5=never done

2.2. Waste management

2.2.1. What measures do you carry out to endeavor proper management of wastes in your premises?

a. Wastes (un-edible parts) are placed in containers with suitably fitted lids and removed frequently from meat handling areas where it is produced. 1= Yes; 2=No
b. Use of waste containers but without lids. 1= Yes; 2=No
c. Waste containers regularly cleaned and disinfected. 1=Yes; 2=No
d. No waste control plan. 1= Yes; 2=No

2.3. Cleaning programs (method and regularity of cleaning and disinfecting meat preparation areas as well as machinery and utensils used)

**Item and areas**	**Frequency of cleaning**; 1=after every use; 2=twice a day; 3= daily; 4= weekly; 5=monthly; 6= no standard practice	**Cleaning materials used**: 1= cold water only; 2= cold water and detergents; 3= Hot water, with detergents and other chemicals
Food contact equipment/ utensils such as chopping boards, tongs, cutting tools, hooks, display trays, containers, etc.		
Non-moveable equipment such as work surfaces, wash hand basin, taps, door handles		
Food waste containers and refuse waste bins/area		
Cloths and work clothes		
Refrigerator/chill and freezer		
Dry storage area		
Floors		

2.4. Public oversight

2.4.1. How often does the government authorities visit premises to check for compliance by the butcher shop? 1=Monthly; 2= Quarterly; 3=Every six months; 4=Annually; 5= Never

2.5. Maintenance (premises and machinery)

2.5.1. How often do you carry out general maintenance checks of the premises and the machinery? 1=Weekly; 2=Monthly; 3=Quarterly; 4=Yearly; 5=Only when there is a breakdown of equipment; 6=Never

2.6. Personal hygiene (Hand Washing, use of gloves, protective clothing, Excluding ill staff from duty)

2.6.1. Indicate which of the personal hygiene practice(s) you often carry out.

a. Hand washing. 1=Yes; 2=No
b. Use of protective clothing like gloves. 1=Yes; 2=No
c. Exclusion from work in case of illness. 1=Yes; 2=No
d. Reporting of illness which poses a risk to meat safety, such as skin, nose, throat, stomach or bowel trouble or if they have any infected wounds. 1=Yes; 2=No

2.7. Environmental hygiene

2.7.1. What measures do you do to ensure that the environment you are working on is safe and would not lead to any cross-contamination?

a. Designate the area where meat is stored and ensure restricted entrance by other people. 1=Yes; 2=No
b. Surfaces and floors are smooth, impervious and capable of being thoroughly cleaned and disinfected. 1=Yes; 2=No
c. Meat is stored on hangers to avoid contamination from the floor. 1=Yes; 2=No
d. Other. Specify……………….

2.8. Correct handling, storage & transport

2.8.1. How is the carcass transported from the slaughterhouse to the butcher shop?

a. refrigerated trucks. 1=Yes; 2=No
b. trucks without refrigerators. 1=Yes; 2=No
c. motorcycles (boda-boda). 1=Yes; 2=No
d. by hand. 1=Yes; 2=No
e. other specify…………

2.8.2. where is the meat stored in while at the butcher shop?

a. In refrigerators, freezers, chilled rooms. 1=Yes; 2=No
b. In a guarded glass compartment. 1=Yes; 2=No
c. On hangers. 1=Yes; 2=No
d. On the floor. 1=Yes; 2=No
e. Other. Specify…………

2.8.3. How long does meat stay before its completely sold off? 1= A day; 2=Two days; 3=Three days; 4=Four days; 5=A week

2.9. Staff training

2.9.1. Please indicate which practices you carry out regarding staff training

a. Training for new staff. 1=Yes; 2=No
b. Retraining. 1=Yes; 2=No
c. HACCP based training. 1=Yes; 2=No
d. Monitoring/checking and any other appropriate records used by your business. 1=Yes; 2=No

2.9.2. Does the government or NGO provide any form of training to your staff? 1=Yes; 2=No

2.9.3. If yes, how often is it provide?

……………………………

2.9.4. Does the government set any specific qualifications to be able to run or open a butcher shop?

a. Yes
b. No

If yes, specify

………
